# Quantitative T1-maps delineate myocardium at risk as accurately as T2-maps - experimental validation with microspheres

**DOI:** 10.1186/1532-429X-13-S1-O62

**Published:** 2011-02-02

**Authors:** Martin Ugander, Paul S Bagi, Abiola J Oki, Billy Chen, Li-Yueh Hsu, Anthony H Aletras, Saurabh Shah, Andreas Greiser, Peter Kellman, Andrew E Arai

**Affiliations:** 1 National Institutes of Health, Bethesda, MD, USA; 2 Siemens Medical Solutions, Chicago, IL, USA; 3 Siemens AG Healthcare Sector, Erlangen, Germany

## Introduction

Clinical quantitative T1 and T2 mapping sequences are available, but it is unclear which, if either, is more accurate for determining myocardium at risk.

## Purpose

To validate and determine the accuracy for using in vivo T1 mapping or T2 mapping for determining myocardium at risk, compared to blood flow quantification at coronary occlusion by microspheres.

## Methods

Dogs (n=12) underwent coronary occlusion (2 hours), during which microspheres were injected into the left atrium, followed by reperfusion (4 hours). The entire left ventricle was imaged at 1.5T (Siemens) in contiguous short-axis slices with a Modified Look-Locker Inversion-recovery sequence for T1 mapping (Messroghli, JMRI, 2007), and a T2-prepared SSFP sequence for T2 mapping (Giri, JCMR, 2009). Myocardium at risk was defined as regions in the left ventricle which had a T1 or T2 value greater than 2SD from remote. In four dogs, hearts were excised and blood flow (ml/min/g) was determined by microsphere analysis in 16 sectors per short-axis slice for all short-axis slices in the left ventricle. Myocardium at risk was defined as regions in the left ventricle with a blood flow at occlusion less than 2SD from remote.

## Results

**Figure 1:**
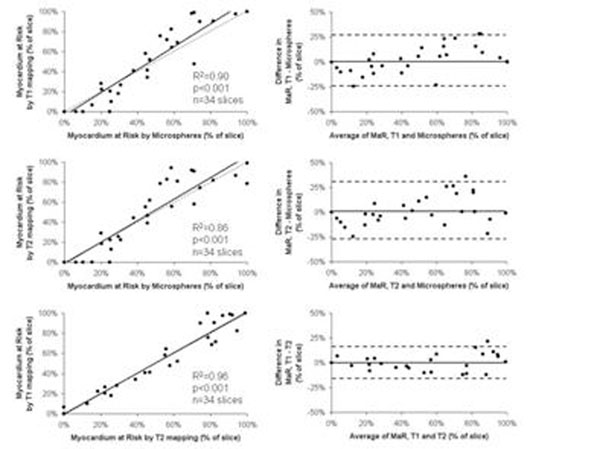
Linear regression (dashed line is line of identity) and Bland-Altman analysis (mean +/- 2SD) for myocardium at risk by T1 mapping vs. T2 mapping vs. microspheres.

Global myocardium at risk, expressed as percent of left ventricular mass (%LVM), showed similar results for T1 mapping compared to T2 mapping (n=12, mean+/-SD difference 1.5+/-4.0 %LVM, R2=0.86, p<0.001). On a slice-by-slice basis (n=4 dogs, 34 slices), myocardium at risk was also similar for T1 and T2 mapping (difference 0.4+/-7.6 % of slice, R2=0.96, p<0.001), T1 mapping corresponded to microsphere analysis (mean difference 2.0+/-12.8 % of slice, R2=0.90, p<0.001), and T2 mapping corresponded to microsphere analysis (mean difference 1.6+/-13.8 % of slice, R2=0.86, p<0.001). Figure 1.

## Conclusions

When it comes to determining myocardium at risk after coronary ischemia and reperfusion, clinical non-contrast T1 mapping and T2 mapping sequences yield similar results, and both correspond excellently to microspheres. It appears that the relaxation properties T1 and T2 both change in a way which is consistent with the myocardial edema which occurs following myocardial ischemia/reperfusion.

